# The Relation between Polyaromatic Hydrocarbon Concentration in Sewage Sludge and Its Uptake by Plants: *Phragmites communis*, *Polygonum persicaria* and *Bidens tripartita*

**DOI:** 10.1371/journal.pone.0109548

**Published:** 2014-10-13

**Authors:** Barbara Gworek, Katarzyna Klimczak, Marta Kijeńska

**Affiliations:** 1 Institute of Environmental Protection – National Research Institute, Warsaw, Poland; 2 Warsaw University of Life Sciences – SGGW, Department of Soil Environment Sciences, Warsaw, Poland; CNRS, France

## Abstract

The aim of the study was to define the relationship between the concentration of PAHs in sewage sludge at a particular location and their amount in various plant materials growing on it. The credibility of the results is enhanced by the fact that sewage sludge from two separate sewage-treatment plants were selected for their influence on the content of PAHs in three plant species growing on them. The investigations were carried out for a period of three years. The results demonstrated unequivocally that the uptake of PAHs by a plant depended on polyaromatic hydrocarbon concentration in the sewage sludge. The correlation between accumulation coefficient of PAH in a plant and the content of the same PAH in the sewage sludge had for three-, four- and five-ring hydrocarbons an exponential character and for six-ring hydrocarbons was of a linear character. The accumulation coefficients calculated for three-ring aromatics were several times higher than for four-ring PAHs; further the coefficient values calculated for five-ring PAHs were several times lower than for four-ring hydrocarbons. Finally, the accumulation coefficient values of six-ring PAHs were the lowest in the series of studied polyaromatic hydrocarbons.

## Introduction

Polycyclic aromatic hydrocarbons (PAHs) are environmental contaminants existing in nature as a complex mixture. They may originate from natural sources, as non-anthropogenic burning of biomass, high temperature thermolysis of organic matter and diagenesis of sedimentary materials. They would be also formed in biosynthetic processes occuring in microorganisms or plants [1]. The concentration of PAHs derived from natural processes is insignificant in comparison with that produced as a result of human activity such as coke and steel manufacturing, waste incineration, domestic heating or transport [2]–[7]. PAHs constitute the largest class of potential carcinogens, to which mutagenic activity is also ascribed [8]. The most studied and most known compound of the group is benzo[a]pyrene [9] Numerous data illustrate the distribution of concentration of PAHs in the atmosphere in areas of smoke emission and in the vicinity of the large urban centers [10]–[12]. Because of their mutagenic and/or carcinogenic properties PAHs are a risk to human health.

It has been confirmed [13]–[14] that the respiratory and dietary exposure to PAHs, placed in the 9^th^ position on the 2011 ATSDR (Agency for Toxic Substances & Disease Registry) [15] substance priority list, may lead to different forms of cancer. PAHs are adsorbed from the atmosphere by aerosol particles, through which they find their way to water, soil and plant foliage during rains. This route is therefore considered appropriate for biomonitoring [16]–[19]. PAHs may accumulate in the organisms due to their low solubility and high octanol-water partition coefficient and undergo long-range transport [20]. PAHs accumulated in vegetables could make them possibly carcinogenic for humans [21]. In order to control this problem, a proper understanding of the mechanism of PAHs uptake by specific plants is important. On the other hand PAHs found in plants could also originate from intrinsic biochemical transformations. Therefore much research has to be done in order to accurately specify the main source of PAHs in plant material. In an attempt to understand the role of the listed causes of PAHs contamination in plant material (mass transfer between atmosphere and plants, ground and roots as well as biosynthesis) studies have been made to evaluate precisely the relation between the amount of PAHs and its content in plants growing on particular bed. Two possible mechanisms for the uptake of PAHs by plants have been suggested. According to the first, the transfer of PAHs from polluted atmosphere to plants occurs via a particle-phase deposition on the waxy leaf or from the gas phase through the stomata [21]–[23]. In the second mechanism, an uptake by the root from the soil solution and then translocation to shoots by liquid phase transfer in the transpiration streams is proposed [21]–[27]. Since the mid 1980s the legislation of the European Union has been focused on the utilization of sewage sludge in agriculture as a natural fertilizer containing especially substantial amount of phosphorus (up to over 12 wt%) and over 7 wt% of nitrogen (e.g. the Directive 86/278/EEC). Due to the accumulation in the plant's leaves (including vegetables) of polyaromatics, showing harmful effects on human health, the determination of the mechanism of their uptake by plants is of the highest importance.

The aim of this study was to determine the correlation between PAHs content in municipal sewage sludge and PAHs uptake by plants growing on them as well as the relationship between PAHs molecular structure and its ability of accumulation in plant.

Sewage sludge from two separate sewage-treatment plants were selected for their influence on the content of PAHs in three plant species growing on them. The investigations were carried out for a period of three years.

## Materials and Methods

### 1. Sampling

Two municipal sewage sludge lagoons were selected for these studies. Samples of the sewage sludge and of the plants growing upon them were collected at each site for a period of 3 years. All plants were self-sown, for the comparison study three plant species (*Phragmites communis*, *Polygonum persicaria* and *Bidens tripartita*) growing in both locations have been chosen. The concentration of thirteen PAHs on the US EPA specification list was determined in the sewage sludge as well as in the plant materials.

### 2. Characteristics of sampling sites and locations

No specific permissions were required for both locations. We had oral permissions to enter to the area belonging to sewage treatment plant. The permission to access land and take samples of sewage sludge and plants was provided by heads of both sewage treatment plants (Białystok, Wodociągi Białostockie and Ryki, Zakład Wodociągów i Kanalizacji). The field studies did not involve endangered or protected species. Specific location of study:

L1 23°10′E 53°08′N

L2 21°57′E 51°37′N

**Location 1 (L1).** Municipal sewage plant in a town with a population of about 290 000 inhabitants and textile, metal, electronic, machine-building, building materials, glass, wood and food industries. The sewage plant was used mainly for the treatment of household wastes. Samples were collected over a period of 3 years from 4 sampling fields each with a surface area of ca. 5000 m^2^.

**Location 2 (L2).** Municipal sewage plant in a small town with a population of 11 000, treating household and small-scale industry wastes and sewage.

The sludge from the communal sewage-treatment plant was stored in four lagoons, each of a surface area of 1500 m^2^. Samples of sewage sludge and plant materials were collected from each sampling site for a period of 3 years. The sewage sludge from each lagoon was collected separately, thereby avoiding the analysis of mixed samples.

The dominating as well as the accompanying plant species were selected for sampling. The intention in these studies was to collect the same plant species.

### 3. Methods

The samples for analysis were dried at room temperature (below 20°C), transferred either to a laboratory mill (plant samples) or ground in a mortar and then fractioned in a sieve of 1 mm mesh (sludge samples).

#### PAHs analyses

The composition of the studied polyaromatic hydrocarbons was determined by high pressure liquid chromatography (HPLC) using Waters chromatograph with photodiode (PDA) and fluorescence (FLSC) detector. The analyses were carried out according to the ISO 13877 method (1998). The identification of the PAHs was based on retention time and UV spectra using capillary column (SUPELCOSIL LC-PAH C18, S-5 µm, 15 cm×4.6 mm) and the quantification of all the investigated PAHs was obtained using signals (pik area) and the calibration curve method. Detection limit of the method was determined at 0.003 ng·g^−1^ for all investigated PAHs analysed together at the same time. Average relative standard deviation for chromatographic analysis was 10% and average expanded uncertainty of method with 95% confidence interval multiplied by the coverage factor k = 2 was less than 30%.

The following procedure was applied for the analysis of sewage sludge and plant material samples: 10–20 g of dried and ground material was mixed with 50 cm^3^ of methylene chloride and extracted in the presence of 1 g of metallic copper in Soxtec apparatus for 5 hrs (boiled for 3.5 hrs and subsequently washed for 1.5 hrs); The extracts were evaporated in rotary vacuum evaporator to a dry residue which was dissolved in 1 ml n-hexane portions. The mixtures were introduced into10 mm-diameter columns containing silica gel and alumina oxide basic powder in layers of 5 cm each in height (layer of column 10 cm in height). The column was eluted with a gradient using 20 cm^3^ methylene chloride/n-hexane solutions in ratios 1∶3 and 15 cm^3^ methylene chloride/n-hexane solutions in ratios 1∶1. Then the eluate was concentrated to a dry residue under a flux of nitrogen. The residue was dissolved in 1 ml acetonitrile. The volume of injection for chromatograph analysis was 10 µl.

The relationship between the content of various PAHs in the sewage sludge and in the plants growing on them was determined by the molar accumulation coefficient (m.a.c.) according to the following formula:



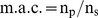



where m.a.c. is molar accumulation coefficient, n_p_ - content of PAHs in a plant material, n_s_ - content of PAHs in sewage sludge.

The corresponding concentration of PAHs, their uptake by plants, as well as accumulation coefficient were expressed as molar magnitudes which allowed their direct further use in kinetic deliberations or biological activity description possessing additive character.

#### Statistical analyses

Relationships between accumulation coefficient of PAH in plant material and the content of PAH in sewage sludge were estimated using simple regression. Linear model (Y = a+bX) or multiplicative model (Y = aX^b^) were selected for the analyses because of good fitting to experimental data. F-test was used for estimation of statistical significance of the examined relationships. The analyses were conducted using Statgraphics 4.1, level of significance was set at 0.05.

## Results and Discussion

In both chosen objects the observations were carried for a period of 3 years and samples were collected in both case from 4 different lagoons. The concentrations of 13 PAHs, listed by USEPA as priority pollutants [28] were estimated:

three-ring (fluorene (Frn), phenanthrene (Ph), anthracene (A))

four-ring (fluoranthene (Ftn), pyrene (P), benzo/a/anthracene (BaA), chrysene (Ch))

five-ring (benzo/b/fluoranthene (BbF), benzo/k/fluoranthene (BkF), benzo/a/pyrene (BaP), dibenz/ah/anthracene (DahA))

six-ring (benzo/ghi/perylene (BghiP), indeno/123-cd/pyrene (IP)).

The determination of the concentrations of naphthalene, acenaphthene and acenaphthylene, was considered not necessary due to their negligible impact - these three compounds are highly volatile [29]. The range of PAHs concentration changes over three years and their average values are presented in the table 1. Fluoranthene occured in highest amounts in the sewage sludge in both locations – an average of 4.19 µmol/kg in location 1 (L1) and 3.14 µmol/kg in location 2 (L2). The second most occurring PAH in the sewage sludge of both locations was pyrene – found in average amounts of 3.09 µmol/kg in L1 and 2.10 µmol/kg in L2. The third PAH in L1 is phenanthrene, which was however in the seventh place in sewage sludge of L2. In both locations three PAHs: fluorene, dibenz/ah/anthracene and anthracene were found in lowest amounts.

**Table 1:** The variability and average content of PAHs.

	The range and average content of PAHs in sewage sludge in both locations [μmol/kg)				The range and average content of PAHs in the foliage of *Phragmites communis* growing on sewage sludge in both locations [μmol/kg]				The range and average content of PAHs in the foliage of *Polygonum persicaria* growing on sewage sludge in both locations [μmol/kg]				The range and average content of PAHs in the foliage of *Bidens tripartita* growing on sewage sludge in both locations [μmol/kg]			
PAH	mean		mean±SD	Range (min-max)			mean±SD	Range (min-max)	mean		mean±SD	Range (min-max)	mean		mean±SD	Range (min-max)
	L1	L2	Both locations		L1	L2	Both locations		L1	L2	Both locations		L1	L2	Both locations	
Frn	0.5	0.34	0.417±0.262	0.003–0.952	0.0963	0.0995	0.098±0.051	0.029–0.209	0.1395	0.1023	0.116±0.055	0.064–0.213	0.1564	0.0729	0.123±0.083	0.022–0.251
Ph	2.32	1.43	1.856±1.04	0.372–4.105	0.2055	0.3411	0.299±0.314	0.137–1.458	0.2713	0.2852	0.28±0.077	0.182–0.416	0.2791	0.3191	0.295±0.063	0.212–0.36
A	0.52	0.30	0.404±0.327	0.109–1.362	0.0163	0.0187	0.018±0.010	0.009–0.036	0.0189	0.0164	0.017±0.007	0.008–0.029	0.0164	0.0228	0.019±0.01	0.01–0.032
Ftn	4.01	3.14	3.558±1.745	0.706–7.043	0.1073	0.1206	0.116±0.025^a^	0.061–0.161	0.1525	0.1584	0.156±0.052^ab^	0.084–0.256	0.1674	0.1736	0.170±0.033^b^	0.136–0.217
P	2.94	2.1	2.503±1.457	0.628–5.671	0.0617	0.0686	0.066±0.025	0.031–0.113	0.0971	0.0721	0.081±0.039	0.041–0.179	0.0875	0.1034	0.094±0.019	0.069–0.114
BaA	1.23	1.2	1.215±0.979	0.225–3.053	0.0050	0.0076	0.007±0.004	0.002–0.015	0.0096	0.0097	0.01±0.004	0.006–0.018	0.0119	0.0100	0.011±0.004	0.007–0.017
Ch	1.64	1.59	1.614±1.214	0.23–3.799	0.0126	0.0214	0.019±0.015	0.007–0.073	0.0273	0.0190	0.022±0.011	0.012–0.049	0.0151	0.0254	0.019±0.009	0.009–0.029
BbF	1.85	2.1	1.981±1.034	0.36–3.801	0.0071	0.0187	0.010±0.004	0.004–0.018	0.0202	0.0130	0.016±0.01	0.008–0.041	0.0082	0.0132	0.01±0.003	0.007–0.014
BkF	0.7	0.91	0.811±0.507	0.153–1.937	0.0034	0.0049	0.004±0.002	0.002–0.007	0.0280	0.0064	0.014±0.025	0.002–0.087	0.0038	0.0054	0.004±0.001	0.003–0.006
BaP	1.52	1.83	1.679±0.924	0.266–3.256	0.0051	0.0073	0.007±0.004	0.002–0.012	0.0133	0.0097	0.011±0.006	0.006–0.026	0.0058	0.0103	0.008±0.003	0.003–0.011
DahA	0.34	0.34	0.342±0.314	0.026–1.163	0.0014	0.0027	0.002±0.001	0.001–0.006	0.0042	0.0021	0.003±0.004	0.000–0.014	0.0023	0.0020	0.002±0.002	0.001–0.004
BghiP	1.47	1.49	1.48±0.764	0.228–2.839	0.0025	0.0057	0.005±0.003	0.001–0.012	0.0096	0.0067	0.008±0.005	0.003–0.018	0.0057	0.0039	0.005±0.002	0.003–0.009
IP	1.2	1.43	1.32±0.691	0.217–2.583	0.0025	0.0050	0.004±0.002	0.001–0.009	0.0092	0.0072	0.008±0.005	0.002–0.018	0.0051	0.0054	0.005±0.001	0.003–0.007

a,b – different letters indicate significant differences between means for species on the basis of Kruskal-Wallis test (P<0.05).

**Figure 1:**
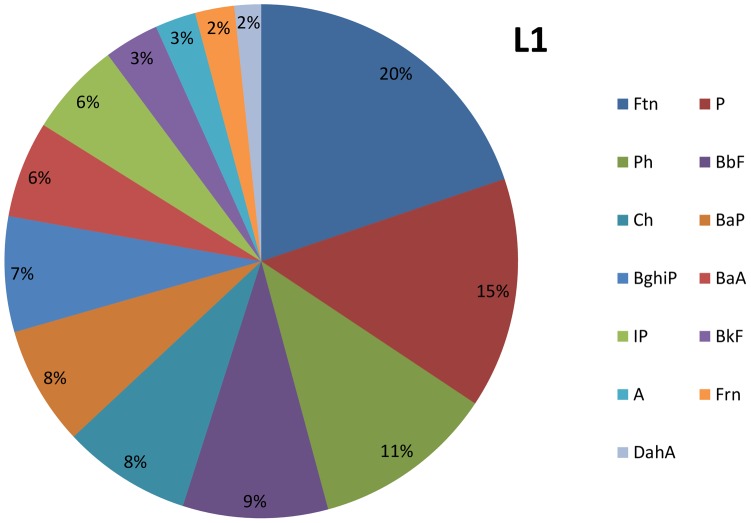
The composition of sewage sludge in L1.

**Figure 2:**
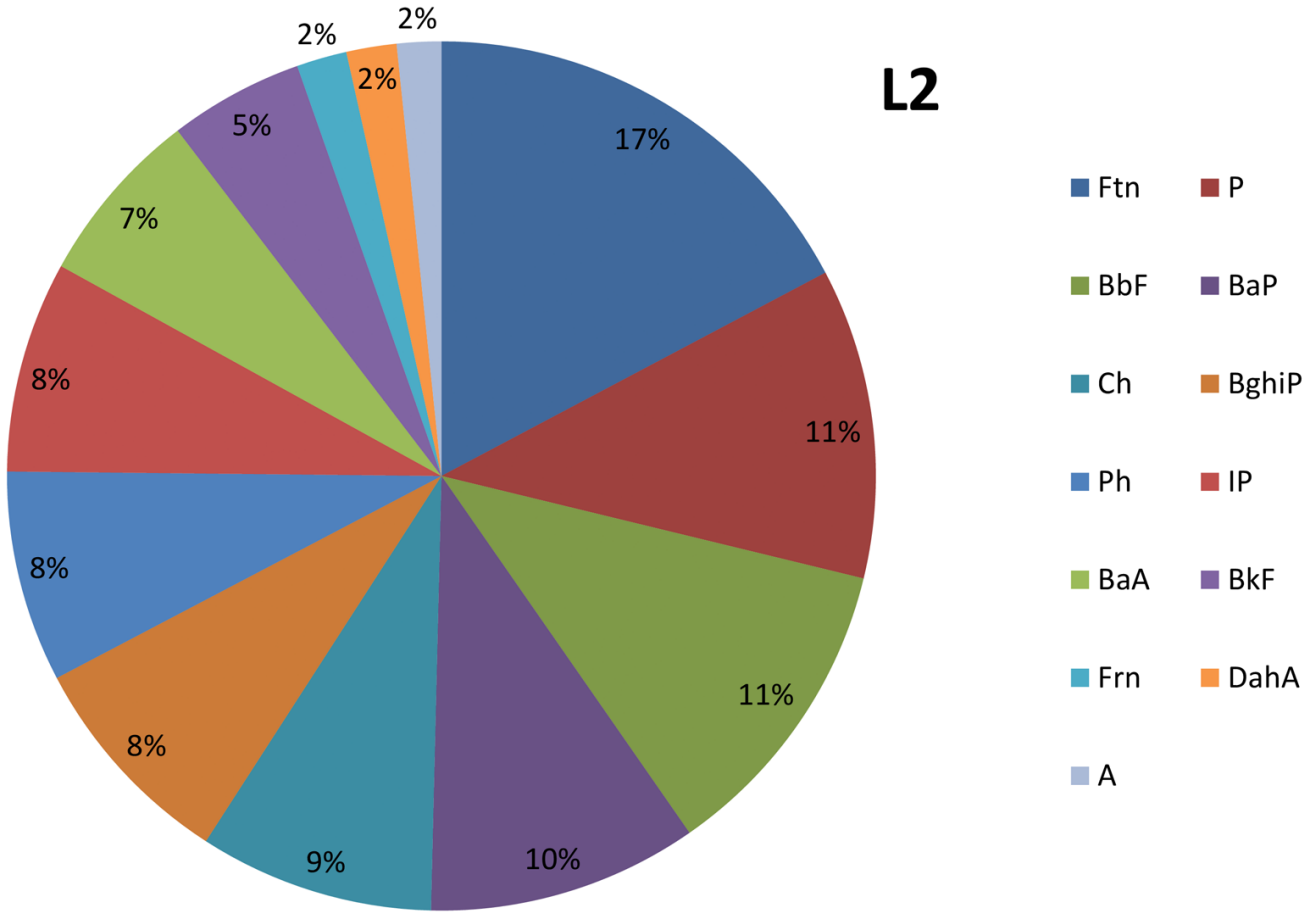
The composition of sewage sludge in L2.

The sequences of PAH concentrations in the sewage sludge are presented below and in figures 1–2.

L1: Ftn>P>Ph>BbF>Ch>BaP>BghiP>BaA>IP>BkF>A>Frn>DahA

L2: Ftn>P>BbF>BaP>Ch>BghiP>Ph>IP>BaA>BkF>Frn>DahA>A

After determining the content of PAHs in sewage sludge, the content of PAHs in the plants growing in them was analyzed. Three plants growing in both locations: common reed (*Phragmites communis*), redshank (*Polygonum persicaria*) and three-lobe beggarticks (*Bidens tripartita*) were analyzed. The shift in the PAH concentration range in each of the plant species during the three years of study and their average values are presented in table 1. Only for Ftn statistically relevant differences between species were stated (between *Phragmites communis* and *Bidens tripartita*). In other cases there were no statistically significant differences observed.

Independently of the content of PAHs in sewage sludge and the species of plant, phenanthrene was a PAH which was present in plant tissue in the highest amount. The content of phenanthrene in the studied plants fluctuated from 0.2055 to 0.3411 µmol/kg (both for *Phragmites communis*). It is worth noting that for each plant species the phenanthrene content was higher in L2, although it occurred in lower amounts in the sewage sludge. The second most abundant PAH in the plants growing in both locations was fluoranthene (0.1073 µmol/kg in *Phragmites communis* collected from L1 to 0.1736 µmol/kg in *Bidens tripartita*, collected in L2). The content of fluoranthene in all plants was also higher in L2, where its amount in the sewage sludge was lower. Fluorene was the third most abundant PAH (although it was fourth after pyrene in *Bidens tripartita* collected in L2) with concentration fluctuating from 0.0729 µmol/kg to 0.1564 µmol/kg. In each case dibenz/ah/anthracene occurred in lowest amounts in the plants (from 0.0014 µmol/kg in *Phragmites communis* collected in L1 to 0.0042 µmol/kg in *Polygonum persicaria* collected in L1).

A comparison of the contents of the studied PAHs in plants did not reveal any clear relationship between the PAH structure and its concentration in plant, probably due to the remarkably varying content of PAHs in the sewage sludge of the various locations. Therefore it was decided to plot the accumulation coefficients which are more objective factors than the content. When the accumulation coefficients for various hydrocarbons are compared, the regularity becomes clear: the uptake efficiency for all plants in both locations under study was several times higher for three-ring PAHs than for four-ring PAHs and, respectively, for four-rings PAHs than for five-ring and six-ring molecules.

**Figure 3:**
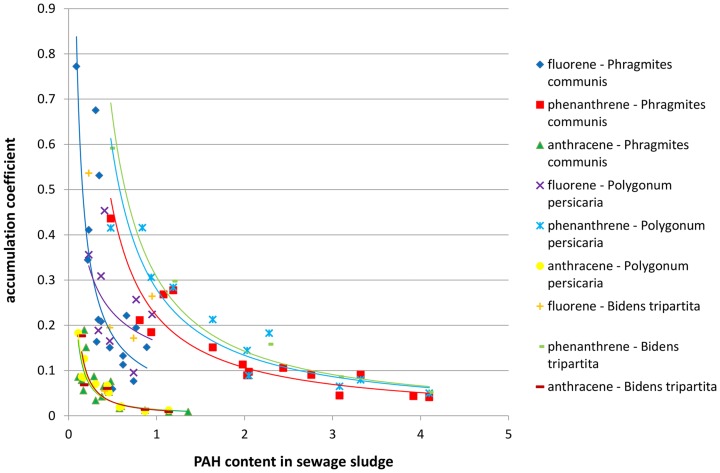
The profiles of dependence: accumulation coefficient of PAH in plant material vs. the content of PAH in sewage sludge for three-ring hydrocarbons.

**Figure 4:**
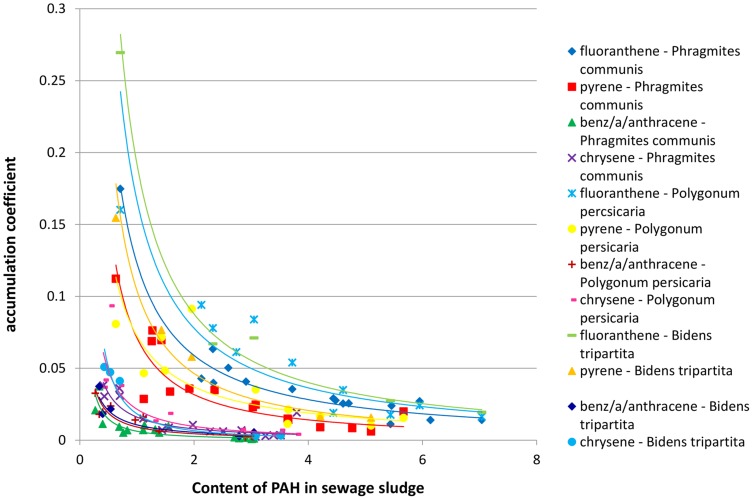
The profiles of dependence: accumulation coefficient of PAH in plant material vs. the content of PAH in sewage sludge for four-ring hydrocarbons.

**Figure 5:**
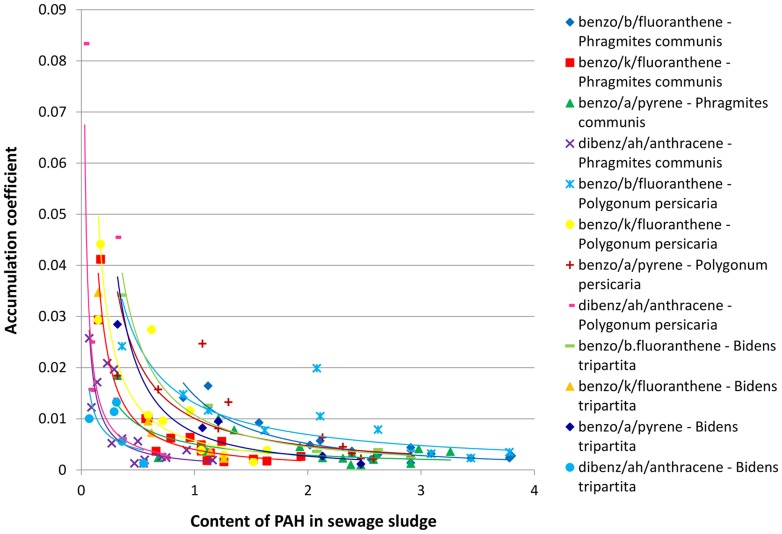
The profiles of dependence: accumulation coefficient of PAH in plant material vs. the content of PAH in sewage sludge for five-ring hydrocarbons.

**Figure 6:**
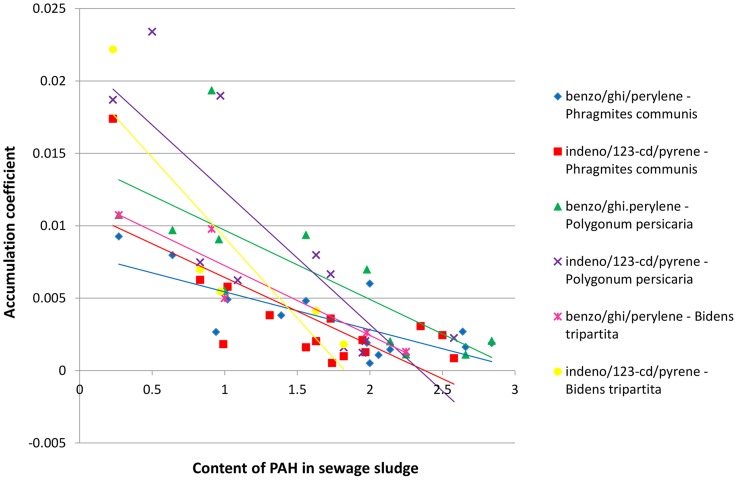
The profiles of dependence: accumulation coefficient of PAH in plant material vs. the content of PAH in sewage sludge for six-ring hydrocarbons.

In order to establish the existence of a regularity in the dependence between the concentration of polyaromatics in sewage sludge and the PAH content in plants growing upon the studied sewage sludge, the relationships between the accumulation coefficient and the content of PAH in the sewage sludge was plotted for three-, four-, five- and six-ring hydrocarbons jointly for both studied locations (Fig. 3–6). It was assumed that a general rule can be developed that would confirm the uptake of polyaromatics from the sewage sludge by plants depending on the amount of PAHs. The results presented in Fig. 3–6 indicated the existence of a correlation between the content of PAHs in sewage sludge and the plant material collected from it. The obtained relationships of accumulation coefficient vs. PAH content in sewage sludge exhibited a different character for various classes of PAHs. It should be emphasized that the data depicted on corresponding Figures (Fig. 3–5) possess high convergence level (Tab. 2) in connection with the fact that they were collected for different plants and various locations at various time, made them reliable. As it is shown in the table 2, most of the correlations between accumulation coefficient of PAH in plant material and the content of PAH in sewage sludge were strong (high coefficient of determination – R^2^) and statistically significant, because the value P<0.05. Only few P values were higher then 0.05.

**Table 2:** Results of regression analysis between accumulation coefficient of PAH in plant material (Y) vs. the content of PAH in sewage sludge (X).

	*Phragmites communis* (n = 16)*				*Polygonum persicaria* (n = 11)				*Bidens tripartita* (n = 5)			
	a	b	R^2^	P	a	b	R^2^	P	a	b	R^2^	P
three-ring hydrocarbons – parameters of regression model: Y = aX^b^												
fluorene	0.095	−0.904	52.2	0.002	0.164	−0.478	22.2	0.239	0.153	−0.320	4.5	0.731
phenanthrene	0.221	−1.055	87.3	<0.001	0.280	−1.067	86.4	<0.001	0.307	−1.105	95.7	0.004
anthracene	0.014	−1.127	72.8	<0.001	0.013	−1.186	86.6	<0.001	0.013	−1.270	90.0	0.014
four-ring hydrocarbons – parameters of regression model: Y = aX^b^												
fluoranthene	0.123	−1.061	86.9	<0.001	0.166	−1.097	81.1	<0.001	0.192	−1.129	97.8	0.001
pyrene	0.071	−1.166	78.5	<0.001	0.072	−0.969	72.2	<0.001	0.104	−1.166	97.5	0.002
benzo/a/anthracene	0.006	−1.244	80.5	<0.001	0.009	−1.036	89.3	<0.001	0.011	−0.953	89.4	0.015
chrysene	0.016	−1.028	69.2	<0.001	0.022	−1.167	88.4	<0.001	0.018	−1.472	97.9	0.001
five-ring hydrocarbons – parameters of regression model: Y = aX^b^												
benzo/b/fluoranthene	0.015	−1.499	79.4	<0.001	0.013	−0.918	63.8	0.003	0.011	−1.250	95.53	0.004
benzo/k/fluoranthene	0.004	−1.195	82.9	<0.001	0.006	−1.142	75.1	0.001	0.004	−1.208	96.2	0.003
benzo/a/pyrene	0.005	−0.810	40.3	0.008	0.010	−1.089	69.9	0.001	0.007	−1.442	88.0	0.018
dibenz/ah/anthracene	0.002	−1.008	63.1	<0.001	0.002	−0.962	63.9	0.006	0.003	−0.679	30.4	0.335
six-ring hydrocarbons – parameters of regression model: Y = a+bX												
Benzo/ghi/perylene	0.008	−0.003	59.3	<0.001	0.014	−0.005	55.7	0.008	0.012	−0.005	86.7	0.021
indeno/123-cd/pyrene	0.011	−0.005	52.7	0.002	0.021	−0.009	69.3	0.002	0.020	−0.011	77.2	0.049

*- in some cases sample size was lower because of missing data.

(a-intercept, b- regression coefficient, R^2^- coefficient of determination, P – observed significance level for F-test, value below 0.05 means significant correlation).

As shown in Figs. 3–5 for PAHs, containing three, four or five aromatic rings, the relationship between the accumulation coefficient and PAH content in sewage sludge was of an exponential character while in the case of molecules containing six aromatic rings (Fig. 6) a linear character of the discussed relationship was undoubtly observed. The exponential function depicting the accumulation coefficient vs. the PAH content in sewage sludge was of y  =  ax^−b^ character i.e. the highest accumulation coefficient value corresponding to the PAH lowest content in the sewage sludge rapidly diminished with an increase of PAH concentration and after which the decrease was insignificant after reaching a specific value for each PAH. This specific level corresponded to much higher values of accumulation coefficient for three-ring aromatics than for four-ring hydrocarbons and, respectively, for five-ring molecules. It is noteworthy that for all the discussed PAH groups the profiles depicting anthracene and its derivatives (benz/a/anthracene and dibenz/ah/anthracene, correspondingly) differed significantly from those ascribed to other PAHs, especially for very low accumulation coefficient values.

For the six-ring PAHs the relationship between the accumulation coefficient and PAH content in sewage sludge was of linear character. The accumulation coefficient values, lower than those calculated for the five-ring PAHs, diminished proportionally with the increase of PAHs content in sewage sludge.

In contrast to the anthracene structure to which a limiting effect in the accumulation susceptibility of polyaromatic hydrocarbons can be ascribed, the isomeric phenanthrene structure seemed to facilitate the accumulation of PAHs. This is shown in the examples of phenanthrene, anthracene, benzo(k)fluoranthene and benzo(b)fluoranthene. Anthracene and phenanthrene are PAHs with a three-ring structure. The linear anthracene molecule should penetrate the plant structure more easily when considering only the physical character of PAH uptake. The phenanthrene molecule possesses a more complex spatial structure, nevertheless, practically in all studied cases its accumulation coefficient was ca. twice higher as compared with those determined for anthracene, and the absolute magnitude of its content was of a range higher than the value established for anthracene. The same PAH uptake behaviour was observed for the benzo/b/fluoranthene and benzo/k/fluoranthene molecules. The structural differences in the last hydrocarbon pair lie in the existence of the so called “bay region” responsible for the carcinogenic activity in benzo[b]fluoranthene molecule [30].

The remarkable differences in the uptake of very similar PAHs in plant materials could be considered as an indirect proof of a specific biochemical pathway of their collecting by plants.

The existence of dependence between the plant accumulation potential for given PAH and the content of this PAH in a ground suggested the existence of some hydrocarbon molecule transfer from sewage sludge to a plant.

In spite of the differences in the character of the studied relationship noted for three-, four- and five-ring PAHs, they all exhibited a diminishing character.

## Conclusions

The results of the present study carried out on three different plant species growing on two independent localizations demonstrated unequivocally that:

- a correlation exists between the content of PAHs in the plant and in sewage sludge;

- this correlation is of an exponential character for three-, four- and five-ring hydrocarbons and of a linear character for six-ring PAHs;

- the observed relationship does not depend on the plant species;

- the appearance of such correlation unequivocally indicated the existence of a path of PAHs uptake by plants from the soil bed;

- the accumulation coefficients calculated for three-ring aromatics were several times higher than for four-ring PAHs; moreover, the coefficient values calculated for five-ring PAHs were several times lower than for four-ring hydrocarbons; finally, the accumulation coefficient values of six-ring PAHs were the lowest in the series of studied polyaromatic hydrocarbons;

- the anthracene derived series of hydrocarbons exhibited lower accumulation coefficient values than other hydrocarbons of the same ring number;

- in contrast, the phenanthrene series was characterized by high accumulation coefficient values.
